# Contributions of clinical simulation in the care of women in situations of violence for training

**DOI:** 10.1590/0034-7167-2024-0402

**Published:** 2025-09-01

**Authors:** Daniella Yamada Baragatti, Letícia da Silva Scotto, Ana Paula de Miranda Araújo Soares, Caroline Izabela Silva, Isabela Martins Gabriel, Claudia Adão Alves, Fernanda Berchelli Girão, Diene Monique Carlos

**Affiliations:** I Universidade Federal de São Carlos. São Carlos, São Paulo, Brazil; II Universidade de São Paulo. Ribeirão Preto, São Paulo, Brazil

**Keywords:** Intimate Partner Violence, Gender-Based Violence, High Fidelity Simulation Training, Primary Health Care, Teaching., Violencia de Pareja, Violencia de Género, Enseñanza Mediante Simulación de Alta Fidelidad, Atención Primaria de Salud, Enseñanza.

## Abstract

**Objectives:**

to verify the contributions of clinical simulation on Primary Healthcare for intimate partner violence against women, through debriefing, from medical and nursing students’ perspective.

**Methods:**

a qualitative study, carried out with 58 participants, between August and October 2023, at a public university in São Paulo, Brazil. Data collection was mediated by holistic debriefing and analyzed using reflective thematic analysis.

**Results:**

three final topics emerged: Comprehensive approach to care for cases of violence; Intervention strategies; and Importance of simulations in healthcare professional training.

**Final Considerations:**

simulation proved to be an important tool for addressing violence against women in health education, in line with the Sustainable Development Goals, and can act as a facilitating tool for the elimination of all forms of violence against women.

## RESUMEN

**Objetivos:**

verificar los aportes de la simulación clínica en la atención en la Atención Primaria de Salud respecto de la violencia de pareja contra las mujeres, a través del *debriefing*, desde la perspectiva de estudiantes de medicina y enfermería.

**Métodos:**

estudio cualitativo, desarrollado, realizado con 58 participantes, entre agosto y octubre de 2023, en una universidad pública de São Paulo, Brasil. La recopilación de datos estuvo mediada por un informe holístico y se analizó mediante un análisis temático reflexivo.

**Resultados:**

surgieron tres temas finales: abordaje integral de los casos de violencia; Estrategias de intervención; e Importancia de las simulaciones en la formación de profesionales de la salud.

**Consideraciones Finales:**

la simulación ha demostrado ser una herramienta importante para abordar la violencia contra las mujeres en la educación sanitaria, en línea con los Objetivos de Desarrollo Sostenible, y puede actuar como una herramienta facilitadora para la eliminación de todas las formas de violencia contra las mujeres.

## INTRODUCTION

Intimate partner violence (IPV), which can cause physical, psychological or sexual harm, is the most common form of violence experienced by women worldwide^(1)^. This is a problem that occurs in a widespread and early manner. Over the course of their lives, between the ages of 15 and 49, 27% of women have experienced physical and/or sexual violence by an intimate partner^(2)^.

In Brazil, in 2019, there were 729 cases of violence against women in the family context per day. In other words, every hour 30 women suffer physical aggression; three women are victims of femicide (homicide committed against a female victim for reasons based on domestic and/or intra-family violence, or in cases of discrimination based on the condition of women) every day; one rape is recorded every eight minutes in the country. The Atlas of Violence found a probable increase in domestic violence against women in Brazil, as homicides of women in homes increased by 10.6% between 2009 and 2019, while murders outside the home showed a reduction of 20.6% in the same period^(3)^.

Violence against women can be explained by gender inequality, the patriarchal system, the macho culture and misogyny (hatred or aversion to women). The COVID-19 pandemic has worsened cases due to social isolation, economic impact, work overload for women, stress and other emotional effects, alcohol and drug abuse, and reduced action of services to combat IPV^(4)^.

Women may suffer the consequences of violence for the rest of their lives, long after violence has ended. The World Health Organization cites individual consequences such as risk of injury, depression, anxiety disorders, unplanned pregnancy, sexually transmitted infections, among other problems. There are also impacts on society as a whole, with tangible or intangible costs^(2)^.

In 2015, during the United Nations General Assembly, composed of 193 member states of the United Nations (UN), global goals were established to lead the world onto a sustainable path and with transformative measures, the so-called Sustainable Development Goals (SDGs). Thus, 17 objectives and 169 global goals were defined to be achieved by 2023. Among the goals is to achieve gender equality and eliminate all forms of violence against all women and girls^(5)^.

Healthcare professionals, especially those working in Primary Health Care (PHC), are in a strategic position to help women in situations of violence, since in this place there is greater proximity to the community, which facilitates the identification of cases^(6)^. Therefore, the entire professional training process in health must involve teaching strategies that enable experiences and reflections on the topic.

To this end, it is necessary to look at healthcare professional training in this area, using active and meaningful teaching-learning strategies. Clinical simulation has been identified as an effective educational method for undergraduate health students to learn. According to a systematic review, students had their knowledge stimulated and were motivated to learn^(7)^. With nursing students, simulation has been a teaching strategy constantly used to develop their clinical reasoning^(8)^.

Nursing, criminal justice, and psychology students were exposed to simulation scenarios about human trafficking, sexual violence, and domestic violence. Post-practice discussions demonstrated that students developed skills such as empathy and collaborative care in assisting people in situations of violence^(9)^.

A case study conducted at a university in Costa Rica sought to understand the pedagogical elements necessary for debriefing, one of the stages of a clinical simulation, to promote reflective thinking. The authors found that debriefing is a pedagogical action that contributes to meaningful learning and the development of reflective thinking in nursing, helping students to transfer knowledge to a real-world context^(10)^. Seeking to promote reflective learning in simulation, researchers developed a holistic debriefing guide that included formative and summative aspects of guidance to assist educators in conducting this important stage^(11)^.

Therefore, considering the magnitude and damage caused by violence against women and that its eradication is one of the Millennium SDGs, highlighting the importance of healthcare professionals being prepared to deal with cases of violence, especially in PHC, this study sought to answer the following research question: what contributions can the use of a clinical simulation scenario supported by a holistic debriefing on IPV care against women in the PHC context bring to health students’ teaching-learning process?

## OBJECTIVES

To verify the contributions of clinical simulation on PHC care for IPV against women, through debriefing, from medical and nursing students’ perspective.

## METHODS

### Ethical aspects

The research was approved by the *Universidade Federal de São Carlos* Research Ethics Committee.

### Study design

This is a descriptive-exploratory study with a qualitative approach^(12)^. The study followed the aspects contained in the COnsolidated criteria for REporting Qualitative research (COREQ).

### Methodological procedures

A clinical scenario was constructed and validated in appearance and content by 26 judges who were experts in clinical simulation and violence against women. Learning objectives were based on Bloom’s Taxonomy, which lists the objectives of educational processes based on the cognitive, affective and psychomotor domains; on the recommended practice standards for simulation design from the International Nursing Association for Clinical Simulation and Learning^(13)^; on a script for constructing a simulated scenario^(14)^; and on the debriefing guide “Three Stages of Holistic Debriefing”^(11)^. A questionnaire, prepared by the researchers, was also used to learn about the socioeconomic profile of participants in simulated scenarios.

Chart 1 describes clinical situation chosen to compose the simulated scenario.

**Chart 1:** Characterization of simulated clinical scenario, São Carlos, São Paulo, Brazil, 2023

Scenario title	Clinical simulation on intimate partner violence against women in PHC
Clinical case	MRL, 30 years old, comes to the Family Health Unit (FHU) complaining of headache, saying that pain is recurrent. The nurse, upon analyzing the patient’s medical records, found that the patient is coming to the FHU for the fourth time this month alone, with complaints of headache and dysuria, and also found that she has recurrent Urinary Tract Infection (UTI). She lives in the Videira neighborhood (a region of high social vulnerability and peripheral) with her husband, CAR, 48 years old, and their 5 children, ages 15, 11, 9, 4 and 2 years old. MRL has incomplete elementary education and reports that she moved to this city when she met her husband and became pregnant with her first daughter, leaving her family in another state and coming here. She works as a domestic worker and, despite her desire, reports that her husband does not want her to work outside the home. She reports using contraceptives and does not remember the name, as her friend recommended that she take them. She says that headache started yesterday around 11 p.m. and even after taking some medication, it did not relieve pain.
Learning objective	Students must be able to develop care for women in situations of intimate partner violence in PHC.
Human resources	An actress (simulated patient).
Material resources	Long dress (to hide bruises on the arms), makeup for mutilation (simulating bruises on the neck), place that characterizes a PHC unit.
Simulation modality	Clinical simulation, with simulated patient.
Stages and duration	Pre-briefing - information booklet (sent 15 days before simulation); Briefing - contract and guidelines (10 to 15 minutes); Clinical simulation (30 minutes); “Three Stages of Holistic Debriefing”^(11)^ (150 minutes).

The woman in a situation of violence was represented by a graduate student, trained to perform this role, aware of learning objectives and with a decision-making flowchart depending on the management of participants during clinical simulation.

### Study setting

The study site was a public university located in the countryside of the state of São Paulo, with a population of 254,857. The university has 64 undergraduate courses and 15,518 students enrolled in 2023. The inclusion criteria for participants were to be a medical or nursing undergraduate student at the university where the study was conducted, to be over 18 years old, to have had contact with theoretical disciplines that dealt with violence and/or women’s health, to be present on the day and at the time agreed for the simulation activity and to participate in all proposed stages. The exclusion criterion was to be away from university activities for any reason. These participants were chosen because they are future higher education professionals who make up the minimum team of the Family Health Strategy.

To invite participants, the research received support for dissemination from the university’s social communication coordination, through emails sent to course coordinators as well as through electronic publication on the platforms and social networks of the university’s academic centers.

Four periods were made available for each undergraduate course, with a maximum of ten vacancies per day. Due to the number of students who contacted the main researcher, three periods were necessary for nursing students and four for medical students. Therefore, those who contacted the researcher and attended the scheduled days for simulation at the Health Simulation Unit, which has simulated environments that are highly similar to PHC offices, participated in the activity.

### Data collection and organization

Simulations with medical and nursing students took place separately, as the intention was not to evaluate comparative differences between the courses, but rather to bring an interdisciplinary perspective.

Clinical simulation took place in stages, with pre-briefing being carried out by sending an information booklet on violence against women^(15)^ 15 days before participation in the scenario. It defined the concept, cycle of violence, prevalence, risk factors and services that are part of the care network, to guide participants’ cognitive knowledge.

On the day of the simulated activity, briefing lasted between 10 and 15 minutes, starting with the presentation of simulated environment and material resources, presentation of facilitators, information to students regarding the learning objective to be achieved in the simulated scenario, in addition to the employment contract, in which all participants were guided on mutual respect, confidentiality and ethics.

Students answered a questionnaire about their socioeconomic profile, received the Informed Consent Form and clinical case description, which they read together with the group. Students were informed that if they did not agree to participate in the research, they could still participate in clinical simulation.

Subsequently, students were invited to volunteer in pairs to participate in the simulated scenario. The remaining students remained as spectators of simulation in another environment - a video room with audio and image systems, in the Health Simulation Unit itself. The scenarios were managed by two facilitators who were experts in the subject and had a flowchart to help conduct the activities, seeking to achieve the learning objectives initially proposed.

Debriefing was recorded using a voice recording application for mobile devices and transcribed manually. The discussion was conducted using the holistic debriefing guide “Three Stages of Holistic Debriefing”^(11)^, covering issues focused on affective, cognitive and procedural learning.

In total, there were seven simulations of IPV against women. Data collection period took place between August and October 2023, with the participation of 28 nursing undergraduate students and 30 medical undergraduate students. Taking into account all stages, the activity with the shortest duration was 2 hours and 35 minutes, and the one with the longest duration, 2 hours and 55 minutes. Chart 2 presents the description of students’ participation in clinical simulation activities.

**Chart 2:** Description of the participation of nursing and medical students in the simulated clinical scenario, São Carlos, São Paulo, Brazil, 2023

	PARTICIPANTS	PRE-BRIEFING	BRIEFING	SIMULATION SCENARIO (resolution)	SIMULATION SCENARIO (observation)	DEBRIEFING	TOTAL
**GROUP 1**	10 nursing students	Whole group	Whole group	2 students	8 students	Whole group	28 NURSING STUDENTS
**GROUP 2**	9 nursing students	Whole group	Whole group	2 students	7 students	Whole group	
**GROUP 3**	9 nursing students	Whole group	Whole group	2 students	7 students	Whole group	
**GROUP 4**	7 medical students	Whole group	Whole group	2 students	5 students	Whole group	30 MEDICAL STUDENTS
**GROUP 5**	8 medical students	Whole group	Whole group	2 students	6 students	Whole group	
**GROUP 6**	8 medical students	Whole group	Whole group	2 students	6 students	Whole group	
**GROUP 7**	7 medical students	Whole group	Whole group	2 students	5 students	Whole group	

For data presentation, participants were identified according to the course they belonged to, being medicine (Med) and nursing (Nur), followed by a sequential number, according to the order in which they spoke during the activity.

### Data analysis

The data were analyzed using the reflexive thematic analysis technique, a method for identifying and analyzing patterns of qualitative data linked mainly to the language used by participants^(12)^. The following stages were adopted: (I) familiarization with the data; (II) coding of relevant information obtained from the extracted data; (III) search for coherent and significant topics for the pattern of relevant data; (IV) review of topics; (V) definition and naming of the three final topics; (VI) final writing.

To ensure greater reliability of this process, the following criteria were followed: (1) use of a field diary that allowed careful and complete recording of the data collection process and inferences in the field; (2) construction of codes and topics between two researchers (1^st^ and 2^nd^ author), agreed upon by a third (last author); (3) checking of analyzed data with participants to verify meaning or the need for new insertions. Moreover, 17 feedbacks were made, with no new information being incorporated.

## RESULTS

Concerning participants’ age, the majority were between 20 and 25 years old (medicine (n=25; 83%), nursing (n=21; 75%)). In relation to race, the majority declared themselves white (medicine (n=22; 73%), nursing (n=19; 68%)). All were cisgender, with 17 of the medicine students identifying as men (57%) and 13 (43%) women. Among the nursing students, three (11%) identified as men and 25 (89%) women.

The results were divided into three final topics presented below.

### Comprehensive approach to dealing with cases of violence

In this regard, it emerged that students from both courses recognize the importance of approaching women in situations of IPV in a comprehensive manner, considering the biopsychosocial aspects and beyond the physical complaints, which are generally the reason for seeking healthcare services. However, in nursing students’ speech, concern with biopsychosocial aspects is more evident:

*But I think that her physical conditions are a result of psychological aggression. I think that we can’t focus only on the physical, or only on issues of violence. I think that there has to be balance. So, as he said, we should gradually address the issue of violence, but we should address it and be open about it.* (Nur 11)

*I think I intended to schedule a follow-up appointment, because, since it was short, we didn’t get to see her biological issues very well, this urinary complaint. We tried to focus more on this social part, to give her time, but I think I would want to schedule a follow-up appointment to have another contact and to address the subject more, as more time went by, to start forming a bond.* (Nur 3)

As highlighted in the last statement, nursing students also recognized the importance of addressing women’s pathophysiological issues. However, medical students demonstrated greater concern with issues such as headaches, self-medication with sedatives, use of contraceptives/possibility of unwanted pregnancy and recurrent urinary infections:

*We tried to address this headache, even though it is nonspecific, probably related to the case, but it is good to characterize it, because it may or may not be a risk. She makes indiscriminate use of contraceptives on her own, so if this headache is with aura, she cannot take some types of contraceptives, for example, as her friend recommended, this part would have to be addressed later, right. The part of self-medication with sedatives would also need to be addressed later. Her recurrent UTI* (Urinary Tract Infection) *would also have to be addressed.* (Med 1)

*Also regarding this issue of her taking sedatives. If they are prescribed, they cannot be used continuously, and that is what seems to be happening, so there would have to be a complete review of what she is using.* (Med 14)

*There is another issue that is more sexual, this issue of contraception, because she can have an unwanted pregnancy, even if the relationships are more superficial, because she just accepts it without there being any desire.* (Med 6)

Despite recognizing the importance of approaching care in a comprehensive manner, students from both courses reported difficulties in managing complex cases with broad health needs:

*She came with complaints. I didn’t know if we should go with the complaints or the other party, and it’s a difficult case to deal with.* (Med 6)

*It’s very difficult, because we have to think about possibilities, provide paths, but in this situation, I don’t know which paths I could contribute to help.* (Nur 2)

There was also, for a nursing student, the fear that care focused on empowering women to learn about and perhaps confront the IPV situation would bring negative consequences for them, instead of helping them:

*A challenge. I felt a bit lost. There are questions that are focused on simulation, but there is also the complaint of pain, so I wanted to bring both things together to be able to provide an answer, a solution. I’m afraid that, as we deconstruct the situation, she’ll end up clashing and the situation will get worse, her husband will end up exploding and this will be yet another stressor, yet another situation for her. But at the same time, I wanted to help in some way, she’s tired, she says, “Oh, because I don’t work and I have to do this”. It’s not quite like that.* (Nur 3)

### Intervention strategies

During simulations, students mobilized and reflected on intervention strategies in the face of the situation presented, such as developing bonds, being concerned about demonstrating the environment safety, developing a formal and informal support network, and providing healthcare for the aggressor.

Students from both courses recognized the importance of empathy when dealing with these cases, considering the sensitivity and complexity of the situations faced by women, demonstrating that it is also necessary to create an environment of trust.

*It ends up being very important to have this flexibility and also the empathy that we need to have when approaching these cases, which are very complex to deal with.* (Med 3)

*Be approachable, because then, for the patient, she will realize that you care.* (Nur 10)

There were considerations about the bond between students from both courses. The importance of respecting women’s wish not to share information about domestic violence experienced in the first contact was also perceived by students as well as the need to build bonds as a tool for care:

*I didn’t see the patient giving any opening to talk about this subject. Every time the girls tried to talk about it, she kind of took it away, so in that first moment, the girls, in my opinion, did very well not to go too deep and just try to bond rather than intrude on what, at that moment, she didn’t want to talk about. At all times, she tried to dodge and didn’t want to talk, so I think it’s time to respect her wishes and create a bond so that, who knows, in a next appointment, she can talk about it.* (Nur 3)

The simulation mobilized and encouraged reflections on care for women in situations of IPV. Students reiterated the importance of supporting these women to understand the situation as violence, but handling it carefully to avoid breaking the bond:

*It would be nice to ask her how she understands this situation. I think that many women who suffer domestic violence do not understand and do not think that they are suffering violence. So, I think it would be nice to ask her if she has ever suffered any type of violence, if she correlates these attacks with violence.* (Med 5)

*So, but we can approach it, like, how to approach this subject? Because you just go like: and that mark on your neck, what is that?* (Nur 1)

*I don’t know if at the beginning I would have said that it was not ideal [how her husband treated her], but I think that, since it was a first appointment and so as not to alienate her, I would maintain this conduct of letting the patient talk, scheduling a next appointment, working on this bond, so that over the course of this bond I can talk about contraceptives, something that I won’t even be able to ask about now.* (Med 12)

Students from both courses identified that women’s support networks, both formal and informal, should be better explored, since they reported that they are distant from their families, identifying that establishing this network is an important tool for care.

*She said that she has no contact with her family. I think this could have been explored further, in the sense of asking why, because this is very normal within the family context of women who suffer violence. I think it would be interesting for us, as professionals, to try to expand her support network, because I think it would be very important for her to reestablish contact, especially because she came from another city, another state, and here she only has her neighbor.* (Nur 16)

Involving the patient’s informal support network, such as friends, neighbors or relatives, can ensure that she has emotional and practical support, and can also be part of a safety plan, including warning signs for the friend, to ensure that she can act in the event of an emergency or need for intervention.

*I was trying to identify some support network, a neighbor, a relative, in case she needed to run, needed to go somewhere, escape.* (Med 1)

*Also talk about the issue of keeping her support network aware, her friend, you know, aware of what she is going through, maybe communicating a sign to her, because it is a bit complicated to take action regarding her husband. She doesn’t work and has 5 children, you know, so letting her friend know about these signs and having a plan B there. I think that’s it, I was trying to get to the part about sexual violence.* (Med 12)

The PHC unit is perceived as an important point in the formal support network for women, since they recognize that they have sought out the unit several times, even if they have not directly reported the situation of violence they have experienced:

*We also see that she went to the unit several times with a headache, so it’s a way for her to seek help. I think this also sends a warning sign.* (Med 29)

*Women sometimes try to seek help through a health center, even though she hasn’t spoken directly, she has been going to the unit several times. She isn’t speaking clearly, but she realizes that what is happening is wrong.* (Nur 13)

*It’s a very difficult situation, so I think I would do the same. One thing that caught my attention is that this is her fourth visit, so she* [...] *is possibly looking for a support network* [...] *she needs to open up to a support network and the health unit has to have this close contact with the patient.* (Med 15)

Students recognized elements of the PHC work process as powerful tools for helping patients, such as involving other team members and home visits. Furthermore, students suggested mapping and informing patients about resources available in the community, such as legal assistance, social services, and shelters for women in situations of violence, highlighting the need for guidance and empowerment to seek help.

*Hers can be addressed in primary care, so ask for a visit from a CHW, they tried to identify a support network to guide her and talk to her. In the end, it would also be interesting to know the entire network available in the municipality, judge, social worker, some housing that shelters women, so she can have a plan. Advise her that this could get worse and have this attitude, never victimize a woman and always show her that she can get out of it. You can see that she is attached, perhaps afraid of what will happen to her son, even her husband, if he is arrested, right, what will happen to her in relation to work?! And this work thing is also interesting, in the definition of violence, preventing her from working is also a type of violence because he is restricting her freedom.* (Med 14)

*Try to talk later that she is experiencing a situation of violence, which involves not only punches, but also holding, shouting, hitting children... also talk and give signs for help, civil police, women’s police station, tell her that this is a welcoming environment for her and her complaints and tell her to reflect on this situation she is experiencing.* (Med 13)

*We could indicate to her, if she confessed to me, services that she might be seeking in relation to this, you know? I think that’s why I’m always very incisive, because I always think about this issue of rescue.* (Nur 22)

Caring for the perpetrator of violence also emerged as a strategy for intervention and care in the face of violence. Participants in the nursing course highlighted the importance of approaching or even treating men, considering that they may be aggressive due to their life history:

*He has been treated like this his whole life and it is natural for him to act this way, you know? Many times, we already see the husband as the aggressor, the one who beats him, the one who hits him, and then we close ourselves off. So, the husband becomes the aggressor, the enemy, and we are there treating the wife as the one being attacked? Sometimes, if the husband goes there, talks to him, opens up and says that what is happening is not ideal, he can change.* (Nur 10)

*Are you going to keep telling this woman, or are you going to make these women go through a relationship with this guy every time, or are we also going to see him as a problem and say, “Look, this is wrong, you need to change your behavior”. So, it’s an issue that needs to be addressed from both sides, right?* (Nur 16)

### Importance of simulations in healthcare professional training

This topic highlighted the importance of simulations for healthcare professional training. According to students from both courses, simulation allowed students to be in touch with feelings, emotions and memories that can interfere with the care provided to women in situations of violence. In the reports below, students shared their own personal experiences with violence, which demonstrates empathy and understanding for the situation of women, in addition to highlighting the lack of support that these people may face, both from healthcare professionals and in other contexts, such as in the family.

*I was just going to share that this was my case too. In general, doctors, at home, nothing, no one ever went into depth. I think it’s an issue that’s very close to my heart, and that makes a big difference, because I never had any support from that side, I faced everything alone, I went through it alone, I ended the relationship alone and that was it. I resolved my issue, but there are many people who can’t.* (Nur 8)

*We have to be very careful because I have already suffered from an abusive and psychological relationship, and violence cannot be justified. People, only God knows how difficult it is to get out of an abusive relationship, it is not easy, not at all, so we have to be careful.* (Nur 14)

The emotional burden that comes with dealing with cases of violence and the need for socio-emotional skills, such as the ability to welcome and understand women’s emotions, were highlighted. The simulation allowed for the discomfort of dealing with such situations to be overcome, seeking to improve these skills:

*It’s a huge emotional burden, you have to have socio-emotional skills to deal with it.* (Med 4)

*I think these scenarios are always uncomfortable, because we can perceive the patient’s discomfort, we can perceive that she is anxious at all times. She doesn’t make eye contact, so this generates anxiety in us.* (Med 23)

Students also highlighted the importance of having contact with these complex cases through simulation before professional practice, since simulations offer a safe and controlled environment to learn and practice, and that dealing with real cases of violence can be emotionally difficult and challenging:

*I think it’s much lighter here than it actually is in practice. Here we know it’s a simulation, but in practice there have already been cases of violence and then you feel a bit lost.* (Med 30)

The following statement highlights the importance of communication skills and dealing with difficult situations when assisting women in situations of IPV, an aspect that was brought to light during the simulation.

*I think it’s more about how to talk, how to get around certain situations, that was the main thing, because we normally don’t have much contact with this part. It’s very difficult for us to be there face to face with the patient and have to deal with these situations that are so stressful for them and that we can physically see that she is discouraged, having physical symptoms. She says she sleeps well, but she’s still very tired, so I think that was the main thing.* (Nur 21)

Students reflected that simulation helps *to expand horizons* (Nur 11) as well as enabling a smoother transition from theory to practice:

*Simulation makes a big difference in any discipline we take, because when we get to practice, we are more prepared to comprehend and understand what is happening. So, I think that every discipline, if possible, should have it, because it makes us more prepared to practice.* (Nur 25)

## DISCUSSION

Students from both courses realized the importance of providing comprehensive care for cases of violence, addressing biopsychosocial issues. They mobilized intervention strategies, such as empathy and establishing an environment of trust through bonding, recognizing the formal and informal support network, and providing healthcare to the perpetrator of violence. Simulation was seen as an important tool in healthcare professional training, allowing students to have contact with the feelings and emotions that these cases bring before professional practice.

Students understood violence as a complex problem that brought many difficulties in how to proceed during care, but they recognized the importance of supporting and envisioning comprehensive care in these situations. Comprehensiveness as one of the principles of the Brazilian Health System^(16)^ brings the premise that healthcare is based on this concept. Hence, it seeks to go beyond curative practice, considering all individual aspects and the social, family and cultural context at all levels of care. There is also dialogue with trauma-centered care, a model that has been discussed especially internationally to indicate paths for care for survivors of violence. It is a person-centered, sensitive model that addresses past or present experiences of trauma^(17)^. It allows people to regain their sense of control and power. It seeks to build on four pillars: recognizing signs and symptoms; accepting the prevalence and impact of trauma; confronting revictimization; and responding with policies and practices aimed at exposure to violence. It is understood that people facing IPV prefer non-judgment, compassion, and confidentiality rather than criticism of their choices or encouragement to leave the situation^(18)^. It is important to highlight that participants in this study reflected and managed the situations based on these aspects.

A literature review on the challenges of providing care for domestic violence from healthcare professionals’ perspective supports our findings. There are barriers related to professionals, such as lack of knowledge and training, lack of time during care, lack of confidence to ask questions, people’s judgment, lack of safety and comfort in asking questions about violence with other professionals nearby^(19)^. For all these reasons, we highlighted the importance of working on the issue of violence against women during undergraduate studies, seeking to provide support for future professionals to deal with these cases in practice.

Similarly, another study conducted with PHC workers on the care of women in situations of domestic violence found that professionals feel insecure when addressing the issue. The need for them to be trained to develop skills to carry out this type of approach was recognized, once again corroborating our findings on the importance of addressing the issue with future professionals^(20)^.

One aspect that was deeply reflected upon by nursing students during simulations was the approach and care required for perpetrators of violence. A Brazilian study sought to verify the effectiveness of the “*Maria da Penha*” Law and concluded that the law contributed to a 43% reduction in hospitalizations related to aggression against women, but did not impact more serious acts of violence, such as injuries related to knives or firearms. This fact may suggest that perpetrators continue to act with their aggressive tendencies after the enactment of the law, i.e., it demonstrates that care for these perpetrators is still a challenge. The article also highlights the importance of establishing empowerment for women to escape situations of violence, in practice, establishing a support network that guarantees the safety of women and their families^(21)^. Concern about these aspects permeated nursing students’ discussions, demonstrating the mobilization of knowledge and maturity in the debate, which were potential for the development of cognitive, attitudinal and procedural skills related to the topic. Given the above, it is considered essential that care for aggressors should also be addressed with medical students, since this topic was not mentioned by them during the simulations.

Still on the subject of care management, medical and nursing students identified the importance of telling women about places or services where they can seek help and the importance of them having a support network. As in our research, recent studies, carried out in various places around the world, highlight the importance of support and social support to improve self-confidence and reduce the effects of violence^(22-24)^.

Many feelings and emotions were reported by students when dealing with cases of violence. In Spain, a study with doctors sought to verify the use of protocols for dealing with IPV. Professionals reported feelings such as fear, frustration or helplessness as obstacles to dealing with this problem, and it is essential that there is training so that professionals’ emotional responses do not have a negative influence on the care provided to women^(25)^. In our findings it became evident that such feelings and emotions can be discussed, exercised and better developed during professional training to favor better practical performance.

Therefore, continuing with the discussion about feelings and emotions, participation in the simulation led some students to report a personal history of experiencing violence. Their speech generally involved the suffering of remembering the cases and the difficulties of not understanding others, including healthcare professionals. Bringing their pain to light during a simulation was an important process for these victims/students to be able to help women in situations of violence in the future, during their professional practice, as they had already had contact with their own feelings when faced with these cases.

Our findings made it clear that, for students, simulations on IPV care against women are important teaching tools in undergraduate health courses, contributing to the approach of the topic and, consequently, leaving professionals better prepared when faced with cases of violence, contributing to the end of all forms of violence against women. Our result is supported by a systematic literature review carried out in 2020, in which simulation emerged as a teaching and learning strategy that is effective in teaching about violence^(26)^.

It is important to highlight the importance of addressing IPV against women in order to achieve the SDGs. A scoping review sought to determine how the SDGs were integrated into higher education worldwide. The authors highlighted how much future professionals need knowledge about the SDGs to guide their practices, with a focus on the determinants of health. However, they found that only a few health education programs explicitly integrated the SDGs, and in relation to the context of gender equality, nothing was found^(27)^. This simulation on IPV care against women in PHC contributes to achieving this goal set by the UN, since these healthcare professionals will be better prepared to deal with these cases, helping women eliminate all forms of violence against them. This technology can be replicated in other contexts, at a low cost and with feasible resources.

Finally, the importance and relevance of the debriefing stage for teaching and learning in nursing was proven through a literature review that analyzed 140 studies related to this topic^(28)^. This finding supports what we found in our research, since our qualitative findings were collected during debriefing and demonstrated the importance and wealth of information, reflections and learning that this stage of simulation provided to students. It is noteworthy that the guide for holistic debriefing proved to be relevant and coherent in approaching this topic, systematically guiding the discussion to achieve the desired objectives.

### Study limitations

The study had limitations. We carried out the activity with students from a public university who voluntarily attended simulation practice, being students in the last years of undergraduate medicine and nursing who carried out the activity in a uniprofessional manner.

We consider it important to carry out simulations of intimate partner violence against women with undergraduate students from other health areas, in a multidisciplinary manner, from public and private universities.

### Contributions to health

The results of this study provide support for addressing IPV against women in PHC, for undergraduate health courses, through clinical simulations. Such technologies are feasible and can be transferred to different contexts. This experience can contribute to the better training of professionals, leading them to be better prepared to concretely address the phenomenon discussed here, in line with the SDG of achieving gender equality and eliminating all forms of violence against women.

## FINAL CONSIDERATIONS

Returning to our initial objective, the clinical simulation contributed to students’ learning process, who recognized the importance of comprehensive care for women in situations of IPV in PHC and of exercising this care through simulation. Knowledge, skills and attitudes were mobilized, developed and exercised for the construction of this care, a facilitating aspect for students to be more capable in the exercise of the profession in the face of the phenomenon worked on.
