# Supplementary material for: Contributions of clinical simulation in the care of women in situations of violence for training
Source: Rev Bras Enferm. 2025 Sep 1;78(Suppl 1):e20240402. doi: 10.1590/0034-7167-2024-0402 (PMC12407877; doi:10.1590/0034-7167-2024-0402)
Supplement: Translation

# Translation: Contribuições da simulação clínica no atendimento à mulher em situação de violência para formação

**DOI:** 10.1590/0034-7167-2024-0402pt

**Authors:** Daniella Yamada Baragatti, Letícia da Silva Scotto, Ana Paula de Miranda Araújo Soares, Caroline Izabela Silva, Isabela Martins Gabriel, Claudia Adão Alves, Fernanda Berchelli Girão, Diene Monique Carlos

**Affiliations:** I Universidade Federal de São Carlos. São Carlos, São Paulo, Brasil; II Universidade de São Paulo. Ribeirão Preto, São Paulo, Brasil

**Keywords:** Violência por Parceiro Íntimo, Violência de Gênero, Treinamento com Simulação de Alta Fidelidade, Atenção Primária à, Saúde, Ensino

## Abstract

**Objetivos:**

verificar as contribuições da simulação clínica sobre atendimento na Atenção Primária à Saúde à violência por parceiros íntimos contra mulheres, por meio de *debriefing*, na perspectiva dos estudantes de medicina e enfermagem.

**Métodos:**

estudo qualitativo, desenvolvido, realizado junto a 58 participantes, entre agosto e outubro de 2023, em uma universidade pública de São Paulo, Brasil. A coleta de dados foi mediada pelo *debriefing* holístico, sendo analisados por meio de análise temática reflexiva.

**Resultados:**

emergiram três temas finais: Abordagem integral no atendimento de casos de violência; Estratégias de intervenção; e Importância das simulações na formação dos profissionais de saúde.

**Considerações Finais:**

a simulação demonstrou ser importante ferramenta da abordagem da violência contra a mulher no ensino da saúde, em consonância com os Objetivos de Desenvolvimento Sustentável, podendo atuar como ferramenta facilitadora para a eliminação de todas as formas de violência contra as mulheres.

## INTRODUÇÃO

A violência por parceiros íntimos (VPI), que pode causar danos físicos, psicológicos ou sexuais, é a forma mais comum de violência experimentada por mulheres no mundo^(1)^. Trata-se de um problema que acontece de maneira generalizada e precoce. Ao longo da vida, entre 15 e 49 anos, 27% das mulheres já experimentaram violência física e/ou sexual por um parceiro íntimo^(2)^.

No Brasil, em 2019, houve 729 casos de violência contra mulheres no contexto familiar por dia. Ou seja, a cada hora, 30 mulheres sofrem agressão física; três mulheres são vítimas de feminicídio (homicídio praticado contra vítima mulher por motivações baseadas em violência doméstica e/ou intrafamiliar, ou em caso de discriminação pela condição de mulher) a cada dia; um estupro a cada oito minutos é registrado no país. No Atlas da Violência, constatou-se um provável crescimento da violência doméstica contra a mulher no Brasil, pois os homicídios de mulheres nas residências cresceram 10,6% entre 2009 e 2019, enquanto os assassinatos fora das residências apresentaram redução de 20,6% no mesmo período^(3)^.

A violência contra a mulher pode ser explicada pela desigualdade de gênero, pelo sistema patriarcal, pela cultura machista e pela misoginia (ódio ou aversão às mulheres). A pandemia de COVID 19 agravou os casos devido ao isolamento social, impacto econômico, sobrecarga do trabalho às mulheres, estresse e outros efeitos emocionais, abuso de álcool e outras drogas, e redução da atuação dos serviços de enfrentamento à VPI^(4)^.

As mulheres podem ter consequências pela violência sofrida pelo resto de suas vidas muito tempo depois da violência ter acabado. A Organização Mundial da Saúde cita como consequências individuais o risco de lesões, depressão, transtornos de ansiedade, gravidez não planejada, infecções sexualmente transmissíveis, entre outros problemas. Há também os impactos na sociedade como um todo, com custos tangíveis ou não^(2)^.

Em 2015, durante a Assembleia Geral das Nações Unidas, composta por 193 Estados-membros da Organização das Nações Unidas (ONU), foram estabelecidas metas mundiais para levar o mundo a um caminho sustentável e com medidas transformadoras, os chamados Objetivos de Desenvolvimento Sustentável (ODS). Assim, foram definidos 17 objetivos e 169 metas globais a serem atingidos até 2023. Entre os objetivos, está previsto alcançar a igualdade de gênero e eliminar todas as formas de violência contra todas as mulheres e meninas^(5)^.

Os profissionais de saúde, com destaque para os que atuam na Atenção Primária à Saúde (APS), estão em posição estratégica para ajudar as mulheres em situação de violência, uma vez que nesse local há uma maior aproximação com a comunidade, o que facilita a identificação dos casos^(6)^. Assim, todo o processo de formação profissional em saúde deve envolver estratégias de ensino que possibilitem vivências e reflexões sobre a temática.

Para tanto, é necessário um olhar para formação de profissionais de saúde nesta área, lançando mão de estratégias de ensino-aprendizagem ativas e significativas. A simulação clínica foi identificada como um método educacional eficaz para aprendizagem dos estudantes de graduação na saúde. De acordo com revisão sistemática, os estudantes tiveram seu conhecimento estimulado e foram motivados a aprender^(7)^. Com estudantes de enfermagem, a simulação tem sido estratégia de ensino constantemente utilizada para desenvolver o seu raciocínio clínico^(8)^.

Estudantes de enfermagem, justiça criminal e psicologia foram expostos a cenários de simulação sobre tráfico de seres humanos, violência sexual e doméstica. As discussões que ocorriam após a prática demonstraram que os estudantes desenvolveram competências como empatia e cuidado colaborativo no atendimento de pessoas em situação de violência^(9)^.

Estudo de caso desenvolvido em universidade da Costa Rica buscou compreender os elementos pedagógicos necessários para que o *debriefing,* uma das etapas de simulação clínica, favoreça o pensamento reflexivo. Assim, os autores constataram que o *debriefing* é uma ação pedagógica que contribui para a aprendizagem significativa e para o desenvolvimento do pensamento reflexivo na enfermagem, colaborando para que estudantes possam transpor o conhecimento para o contexto real^(10)^. Buscando promover a aprendizagem reflexiva na simulação, pesquisadoras desenvolveram um guia holístico de *debriefing* que incluiu aspectos formativos e sumativos de orientação para auxiliar os educadores na condução dessa importante etapa^(11)^.

Destarte, considerando a magnitude, os danos causados pela violência contra as mulheres e que sua erradicação é um dos ODS do milênio, destacando-se a importância dos profissionais de saúde estarem preparados para lidar com os casos de violência, principalmente na APS, este estudo buscou responder à seguinte pergunta de pesquisa: quais as contribuições que o uso de um cenário de simulação clínica apoiado por um *debriefing* holístico sobre o atendimento à VPI contra mulheres no contexto da APS podem trazer para o processo de ensino-aprendizagem de estudantes de saúde?

## OBJETIVOS

Verificar as contribuições da simulação clínica sobre atendimento na APS à VPI contra mulheres, por meio do *debriefing*, na perspectiva dos estudantes de medicina e enfermagem.

## MÉTODOS

### Aspectos éticos

A pesquisa foi aprovada pelo Comitê de Ética em Pesquisa da Universidade Federal de São Carlos.

### Tipo de estudo

Estudo descritivo-exploratório, com abordagem qualitativa^(12)^. O estudo seguiu os aspectos contidos no *COnsolidated criteria for REporting Qualitative research* (COREQ).

### Procedimentos metodológicos

Um cenário clínico foi construído e validado em aparência e conteúdo por 26 juízes especialistas em simulação clínica e violência contra as mulheres. Teve os objetivos de aprendizagem fundamentados na Taxonomia de Bloom, a qual elenca os objetivos dos processos educacionais baseados nos domínios cognitivos, afetivos e psicomotores; nas normas de práticas recomendadas para o *design* da simulação da *International Nursing Association for Clinical Simulation and Learning^(^* ^13)^; no roteiro para construção de cenário simulado^(14)^; e no guia de *debriefing* “Três Estágios do *Debriefing* Holístico”^(11)^. Também foi utilizado questionário, elaborado pelas pesquisadoras, para conhecer o perfil socioeconômico dos participantes dos cenários simulados.

A situação clínica escolhida para compor o cenário simulado encontra-se descrita no Quadro 1.

**Quadro 1:** Caracterização do cenário clínico simulado, São Carlos, São Paulo, Brasil, 2023

Título do cenário	Simulação clínica sobre violência por parceiro íntimo contra mulher na APS
Caso clínico	MRL, 30 anos, comparece à Unidade de Saúde da Família (USF) com queixa de cefaleia, dizendo que as dores são recorrentes. A enfermeira, ao analisar o prontuário, verificou que a usuária está vindo à USF pela quarta vez somente neste mês, com queixas de cefaleia e disúria, verificando também que ela tem Infecção do Trato Urinário de repetição. Reside no bairro Videira (região de alta vulnerabilidade social e periférica) com seu esposo, CAR, de 48 anos e seus 5 filhos, de 15, 11, 9, 4 e 2 anos. MRL possui ensino fundamental incompleto e relata que se mudou para esta cidade quando conheceu seu marido e engravidou de sua primeira filha, deixando sua família em outro estado e vindo para cá. Exerce trabalho doméstico e, apesar do desejo, refere que o marido não quer que ela trabalhe fora de casa. Relata fazer uso de anticoncepcional e não se lembra o nome, pois sua amiga que indicou que ela tomasse. Diz que a dor de cabeça teve início ontem por volta das 23 horas, e mesmo após tomar um medicamento, não aliviou.
Objetivo de aprendizagem	O estudante deverá ser capaz de desenvolver o cuidado à mulher em situação de violência por parceiro íntimo na APS.
Recursos humanos	Uma atriz (paciente simulado).
Recursos materiais	Vestido comprido (esconder hematomas nos braços), maquiagem para mulagem (simulação de hematomas no pescoço), local que caracterize uma unidade de APS.
Modalidade da simulação	Simulação clínica, com paciente simulado.
Etapas e tempo de duração	*Pré-briefing* - cartilha informativa (envio 15 dias antes da simulação); *Briefing* - contrato e orientações (10 a 15 minutos); Simulação clínica (30 minutos); “Três Estágios do *Debriefing* Holístico”^(11)^ (150 minutos).

A mulher em situação de violência foi representada por uma estudante de pós-graduação, treinada para execução deste papel, ciente dos objetivos de aprendizagem e com um fluxograma de tomada de decisão a depender do manejo dos participantes durante a simulação clínica.

### Cenário do estudo

O local de estudo foi uma universidade pública localizada no interior do estado de São Paulo que possui 254.857 habitantes. A universidade possui 64 cursos de graduação e 15.518 estudantes matriculados em 2023. Os critérios de inclusão para os participantes foram ser estudante de graduação de medicina e enfermagem da universidade do estudo, ser maior de 18 anos, ter tido contato com disciplinas teóricas que tratavam da violência e/ou saúde da mulher, comparecer no dia e horários combinados para a atividade de simulação e participar de todas as etapas propostas. Já o critério de exclusão foi estar afastado das atividades universitárias por quaisquer motivos. A escolha desses participantes se deu por se tratar de futuros profissionais de nível superior que compõem a equipe mínima da Estratégia Saúde da Família.

Para o convite dos participantes, a pesquisa contou com apoio para divulgação da coordenadoria de comunicação social da universidade, por meio de e-mails disparados às coordenações dos cursos, assim como pela publicação em meio eletrônico nas plataformas e redes sociais dos centros acadêmicos da universidade.

Foram disponibilizados quatro períodos para cada curso de graduação com, no máximo, dez vagas por dia. Pela quantidade de estudantes que entraram em contato com a pesquisadora principal, foram necessários três períodos para os estudantes de enfermagem e quatro para os de medicina. Dessa forma, participaram da atividade aqueles que entraram em contato com a pesquisadora e compareceram nos dias agendados para a realização da simulação, na Unidade de Simulação em Saúde, que possui ambientes simulados de alta similitude a consultórios de APS.

### Coleta e organização dos dados

As simulações com os estudantes de medicina e enfermagem aconteceram separadamente, pois a intenção não foi avaliar diferenças comparativas entre os cursos, mas sim trazer uma perspectiva interdisciplinar.

A simulação clínica aconteceu em etapas, sendo que o *pré-briefing* ocorreu através do envio prévio de uma cartilha informativa sobre violência contra mulheres^(15)^ 15 dias antes da participação no cenário. Nela, havia definição do conceito, ciclo da violência, prevalência, fatores de risco e serviços que fazem parte da rede de atenção, para balizar o conhecimento cognitivo dos participantes.

No dia da atividade simulada, o *briefing* ocorreu com duração entre 10 e 15 minutos, sendo iniciado com a apresentação do ambiente simulado e recursos materiais, apresentação das facilitadoras, informações aos estudantes quanto ao objetivo de aprendizagem a ser atingido no cenário simulado, além do contrato de trabalho, em que todos os participantes foram orientados sobre o respeito mútuo, confidencialidade e ética.

Os estudantes responderam um questionário sobre seu perfil socioeconômico, receberam o Termo de Consentimento Livre e Esclarecido e a descrição do caso clínico, o qual realizou a leitura em conjunto ao grupo. Os estudantes foram informados que, caso não concordassem em participar da pesquisa, ainda assim poderiam participar da simulação clínica.

Na sequência, os estudantes foram convidados a se voluntariar em duplas para participarem do cenário simulado. Os demais estudantes permaneceram como espectadores da simulação em outro ambiente - uma sala de vídeo composta por sistemas de áudio e imagem, na própria Unidade de Simulação em Saúde. Os cenários foram gerenciados por duas facilitadoras *experts* na temática que dispunham de um fluxograma para auxiliar na condução das atividades, buscando atingir os objetivos de aprendizagem inicialmente propostos.

O *debriefing* foi gravado utilizando um aplicativo de gravação de voz para aparelhos celulares e transcrito manualmente. A discussão foi conduzida por meio do guia holístico de *debriefing* “Três Estágios do *Debriefing* Holístico”^(11)^, contemplando questões com foco nas aprendizagens afetiva, cognitiva e procedimental.

No total, foram sete simulações de VPI contra mulheres. O período da coleta de dados ocorreu entre agosto e outubro de 2023, com a participação de 28 graduandos em enfermagem e 30 em medicina. Contabilizando todas as etapas, a atividade com menor duração foi de 2 horas e 35 minutos, e a com maior duração, 2 horas e 55 minutos. O Quadro 2 apresenta a descrição de participação dos estudantes nas atividades de simulação clínica.

**Quadro 2:** Descrição da participação dos estudantes de enfermagem e medicina no cenário clínico simulado, São Carlos, São Paulo, Brasil, 2023

	PARTICIPANTES	*PRÉ-BRIEFING*	*BRIEFING*	CENÁRIO DE SIMULAÇÃO (resolução)	CENÁRIO DE SIMULAÇÃO (observação)	*DEBRIEFING*	TOTAL
**GRUPO 1**	10 estudantes de Enfermagem	Grupo todo	Grupo todo	2 estudantes	8 estudantes	Grupo todo	28 ESTUDANTES DE ENFERMAGEM
**GRUPO 2**	9 estudantes de Enfermagem	Grupo todo	Grupo todo	2 estudantes	7 estudantes	Grupo todo	
**GRUPO 3**	9 estudantes de Enfermagem	Grupo todo	Grupo todo	2 estudantes	7 estudantes	Grupo todo	
**GRUPO 4**	7 estudantes de Medicina	Grupo todo	Grupo todo	2 estudantes	5 estudantes	Grupo todo	30 ESTUDANTES DE MEDICINA
**GRUPO 5**	8 estudantes de Medicina	Grupo todo	Grupo todo	2 estudantes	6 estudantes	Grupo todo	
**GRUPO 6**	8 estudantes de Medicina	Grupo todo	Grupo todo	2 estudantes	6 estudantes	Grupo todo	
**GRUPO 7**	7 estudantes de Medicina	Grupo todo	Grupo todo	2 estudantes	5 estudantes	Grupo todo	

Para a apresentação dos dados, os participantes foram identificados de acordo com o curso a que pertenciam, sendo medicina (Med) e enfermagem (Enf), seguidas por um número sequencial, de acordo com a ordem em que falaram durante a atividade.

### Análise dos dados

Os dados foram analisados a partir da técnica de análise temática reflexiva, um método para identificar e analisar padrões de dados qualitativos vinculados principalmente à linguagem utilizada pelas participantes^(12)^. Foram adotados os seguintes passos: (I) familiarização com os dados; (II) codificação das informações relevantes obtidas dos dados extraídos; (III) busca por temas coerentes e significativos para o padrão dos dados relevantes; (IV) revisão de temas; (V) definição e nomeação dos três temas finais; (VI) escrita final.

Para maior confiabilidade deste processo, foram seguidos os seguintes critérios: (1) utilização de diário de campo que permitiu cuidadoso e completo registro do processo de coleta e inferências em campo; (2) construção dos códigos e temas entre duas pesquisadoras (1ª e 2ª autora) consensualizados por uma terceira (última autora); (3) checagem dos dados analisados junto aos participantes para verificação de sentido ou necessidade de novas inserções. Além disso, 17 devolutivas foram realizadas, não sendo incorporadas novas informações.

## RESULTADOS

Em relação à idade dos participantes, a maioria possuía entre 20 e 25 anos (medicina (n=25; 83%), enfermagem (n=21; 75%)). Quanto à cor, a maioria se declarou branca (medicina (n=22; 73%), enfermagem (n=19; 68%)). Todos eram cisgêneros, sendo que estudantes de medicina (17) se identificavam como homens (57%), e 13 (43%), mulheres. Já entre os estudantes de enfermagem, três (11%) se identificavam como homens, e 25 (89%), mulheres.

Os resultados foram divididos em três temas finais, apresentados a seguir.

### Abordagem integral no atendimento de casos de violência

Neste tema, emergiu que os estudantes de ambos os cursos reconhecem a importância de abordar a mulher em situação de VPI de maneira integral, considerando os aspectos biopsicossociais e para além das queixas físicas, que são geralmente o motivo da procura pelo serviço de saúde. Porém, na fala dos estudantes de enfermagem, fica mais evidente a preocupação com os aspectos biopsicossociais:

*Mas eu acho que essas condições físicas dela são decorrentes da agressão psicológica. Eu acho que não dá para focar só na física, ou focar só nas questões de violência, acho que tem que ter equilíbrio sempre. Assim, igual ele falou, ir aos poucos na questão da violência, mas abordar e se mostrar aberta assim*. (Enf 11)

*Acho que eu pretendia marcar um retorno, porque, assim, por ter sido curtinho, gente não conseguiu ver muito bem as questões biológicas dela, essa queixa urinaria. A gente tentou focar mais nessa parte social, para dar tempo, mas eu acho que eu iria querer marcar um retorno para ter um outro contato e ir abordando mais o assunto, conforme fosse passando um pouco mais o tempo, para ir formando o vínculo.* (Enf 3)

Conforme destacado na última fala, os estudantes de enfermagem também reconheceram a importância de abordar as questões fisiopatológicas da mulher. Porém, os estudantes de medicina demonstraram mais preocupação com questões como cefaleia, automedicação com calmantes, uso de anticoncepcional/possibilidade de gravidez indesejada e infecções urinárias de repetição:

*A gente tentou abordar essa cefaleia, mesmo que seja inespecífica, provavelmente relacionada ao caso, mas é bom caracterizar, porque pode ou não ser risco. Ela faz uso indiscriminado de anticoncepcional por conta, então essa cefaleia, se for com aura, ela não pode tomar alguns tipos de anticoncepcional, por exemplo, como foi indicação da amiga, tinha que ser trabalhada essa parte depois, né. A parte da automedicação com calmante também precisaria ser trabalhada depois. A ITU* (Infecção do Trato Urinário) *dela de repetição teria que ser vista também.* (Med 1)

*E também em relação a essa questão de ela estar tomando calmante. Se for tarja preta, não se pode usar de forma contínua, e é o que parece que está acontecendo, então teria que ter toda essa revisão do que ela está usando*. (Med 14)

*Tem outra questão também que é a parte mais sexual, essa questão do anticoncepcional, porque ela pode ter uma gravidez indesejada, ainda que as relações estão mais superficiais, porque ela só aceita sem que exista algum desejo.* (Med 6)

Apesar de reconhecerem a importância em abordar o cuidado de forma integral, os estudantes de ambos os cursos relataram dificuldades no manejo de casos complexos e com necessidades de saúde amplas:

*Ela veio com as queixas. Eu não sabia se íamos nas queixas ou na outra parte, e é um caso difícil de atender.* (Med 6)

*É muito difícil, porque a gente tem que pensar em possibilidades, dar caminhos, mas nessa situação, eu não sei quais caminhos eu poderia contribuir para ajudar.* (Enf 2)

Houve também, para um estudante de enfermagem, o receio do atendimento focado em empoderar a mulher para o conhecimento e talvez enfrentamento da situação de VPI trazer consequências negativas para a mesma em vez de ajudá-la:

*Um desafio. Eu me senti meio perdida. Tem as questões que são voltadas para a simulação, mas tem a queixa da dor, então queria trazer as duas coisas para conseguir dar uma resposta, uma solução. Eu tenho medo de que, conforme a gente desconstrua, ela acabe batendo de frente e a situação acabe piorando, o marido acabe explodindo e isso seja mais um estressor, mais uma situação para ela. Mas ao mesmo tempo, eu queria ajudar de alguma forma, ela está cansada, ela fala: “Ai, porque eu não trabalho e tenho que fazer isso”. Não é bem assim.* (Enf 3)

### Estratégias de intervenção

Durante as simulações, os estudantes mobilizaram e refletiram sobre estratégias de intervenção diante da situação apresentada, como o desenvolvimento do vínculo, a preocupação em demonstrar a segurança do ambiente, o desenvolvimento da rede de apoio formal e informal, e os cuidados em saúde para o agressor.

Os estudantes de ambos os cursos reconheceram a importância da empatia ao lidar com esses casos, considerando a sensibilidade e complexidade das situações enfrentadas pela mulher, demonstrando que também é preciso criar um ambiente de confiança.

*Acaba sendo bem importante ter essa flexibilidade e a forma também com a empatia que precisamos ter ao abordar esses casos, que são muito complexos de lidar.* (Med 3)

*Se mostrar acessível, porque aí, para paciente, ela vai perceber que você se importa.* (Enf 10)

Houve considerações sobre o vínculo pelos estudantes de ambos os cursos. A importância de respeitar a vontade da mulher em não compartilhar informações sobre a violência doméstica vivenciada no primeiro contato também foi percebida pelos estudantes, assim como a necessidade de construção do vínculo como ferramenta para o cuidado:

*Eu não vi a paciente dando abertura para entrar nesse assunto. Todas as vezes que as meninas tentavam entrar, ela meio que tirava, aí, nesse primeiro momento, as meninas, no meu ponto de vista, fizeram muito bem de não ir muito afundo e tentar somente o vínculo do que invadir o que, naquele momento, ela não queria contar. Em todo momento, ela tentava se esquivar e não queria falar, então acho que é o momento de respeitar a vontade dela e criar o vínculo para, quem sabe, numa próxima consulta, ela falar*. (Enf 3)

A simulação mobilizou e favoreceu reflexões para o cuidado à mulher em situação de VPI. Os estudantes retomaram a importância de apoiar esta mulher a compreender a situação vivida como violência, mas manejando de forma cuidadosa para evitar quebra de vínculo:

*Seria legal perguntar como ela entende essa situação. Acho que muitas das mulheres que sofrem violência doméstica não entendem e não acham que estão sofrendo violência. Então, acho que seria legal perguntar se ela já sofreu algum tipo de violência, se ela correlaciona essas agressões com violência.* (Med 5)

*Então, mas a gente pode abordar, assim, tipo, como chegar nesse assunto? Porque você só chegar tipo: e essa marca no pescoço, o que é isso?* (Enf 1)

*Não sei se já nesse primeiro momento eu falava que aquilo não era o ideal* [como o marido a tratava], *mas eu acho que, por ser uma primeira consulta e para não afastar, manteria essa conduta de deixar a paciente falar, marcar uma próxima consulta, ir trabalhando esse vínculo, para ao longo desse vínculo conseguir falar sobre o anticoncepcional, coisa que eu não vou nem conseguir perguntar agora.* (Med 12)

Os estudantes de ambos os cursos identificaram que a rede apoio da mulher, formal e informal, deve ser melhor explorada, uma vez que houve o relato de que ela está distante da família, identificando que o estabelecimento desta rede é uma importante ferramenta para o cuidado.

*Ela ter falado que não tem contato com a família. Eu acho que isso poderia ter sido mais explorado, no sentido de perguntar o porquê, porque isso é muito normal dentro de um contexto familiar das mulheres que sofrem violência. Acho interessante nós, como profissionais, tentar ampliar a rede de apoio dela, porque eu acho que seria muito importante ela retomar o contato, principalmente porque ela veio de outra cidade, outro estado, e aqui ela só tem a vizinha.* (Enf 16)

O envolvimento da rede de apoio informal da paciente, como amigos, a vizinha ou parentes, pode garantir que ela tenha suporte emocional e prático, além de poder fazer parte de um plano de segurança, incluindo sinais de alerta para a amiga, para garantir que ela possa agir em caso de emergência ou necessidade de intervenção.

*Estava tentando identificar alguma rede de apoio, uma vizinha, um parente, para caso ela precise correr, precise ir para algum lugar, fugir.* (Med 1)

*Também falar sobre a questão de deixar a rede de apoio dela ciente, a amiga dela, né, ciente do que ela está passando, talvez comunicar com ela um sinal, porque é um pouco complicado tomar uma ação a respeito do marido. Ela não trabalha e tem 5 filhos, né, então deixar essa amiga dela ciente desses sinais e ter esse plano B aí. Acho que é isso, eu estava tentando chegar na parte da violência sexual.* (Med 12)

A unidade de APS é percebida como um importante ponto da rede de apoio formal para a mulher, uma vez que reconhece que ela buscou a unidade diversas vezes, mesmo que não tenha relatado diretamente a situação de violência vivenciada:

*A gente vê também que ela foi várias vezes na unidade com dor de cabeça, então é uma forma dela procurar ajuda, acho que isso traz um sinalzinho também, de alerta.* (Med 29)

*As mulheres tentam, às vezes, através do posto de saúde, elas procuram ajuda, por mais que não falou diretamente, ela está indo várias vezes. Para a unidade, ela não está falando claramente, mas ela percebe que o que está acontecendo está errado.* (Enf 13)

*É uma situação muito difícil, então eu acho que faria igual. Uma coisa que me chamou atenção é que essa é a quarta visita dela, então ela* [...] *possivelmente está procurando uma rede de apoio* [...] *ela precisa se abrir com uma rede de apoio, e a unidade de saúde tem que ter esse contato próximo ao paciente.* (Med 15)

Os estudantes reconheceram elementos do processo de trabalho na APS como ferramentas potentes de auxílio, como o envolvimento de outros membros da equipe e visita domiciliar. Além disso, os estudantes sugeriram mapear e informar à paciente sobre os recursos disponíveis na comunidade, como assistência jurídica, assistência social e abrigos para mulheres em situação de violência, destacando a necessidade de orientação e empoderamento dela para buscar ajuda.

*A dela pode ser trabalhada na atenção primária, então, solicitar a visita de algum ACS, eles tentaram identificar uma rede de apoio para orientar ela e falar para ela. No final, também seria interessante conhecer toda a rede do município disponível, juiz, assistente social, alguma moradia que abrigasse mulheres, para ela ter um plano. Orientar ela que isso poderia se agravar e ter essa postura, jamais vitimizar uma mulher e sempre mostrar que ela pode sair daquilo. Dá para perceber que ela está apegada, talvez com medo do que vai acontecer com o filho, até mesmo com o marido, se ele for preso, né, como é que vai ficar em relação ao trabalho?! E essa coisa do trabalho também é interessante, na definição de violência, ele impedir ela trabalhar também é um tipo de violência porque ele está cerceando a liberdade.* (Med 14)

*Tentar falar depois que ela está vivendo uma situação de violência, que não só soco, mas segurar, gritar, bater nas crianças também... também falar e dar os sinais de ajuda, polícia civil, delegacia da mulher, falar que aqui é um ambiente acolhedor para ela e para as queixas dela e falar para ela refletir sobre essa situação que está vivendo.* (Med 13)

*A gente poderia indicar para ela, caso ela confessasse para mim, serviços que ela poderia estar buscando em relação a isso, entendeu? Eu acho que é por isso que eu sempre sou muito incisiva, porque eu penso sempre nessa questão do resgate*. (Enf 22)

O cuidado ao autor da violência também emergiu como uma estratégia de intervenção e cuidado à violência. Os participantes do curso de enfermagem trouxeram a importância de abordar ou até atender o homem, considerando que ele pode ser agressivo devido à sua história de vida:

*Ele foi tratado assim a vida inteira, e para ele agir dessa forma, é natural, entendeu? Muitas vezes, a gente já vai com olhar de que é o marido o agressor, é aquele que espanca, aquele que bate, e aí a gente se fecha. Então, o marido vira agressor, o inimigo, e a gente está ali tratando a esposa como a agredida? Às vezes, o marido, se a gente for lá, conversar, abrir e falar que isso que está acontecendo não é o ideal, pode mudar.* (Enf 10)

*Vai ficar falando para essa mulher, ou vai fazer essas mulheres passarem por um relacionamento com esse cara todas as vezes, ou a gente também vai enxergar ele com um problema e vai falar: “Olha, isso aqui está errado, você precisa mudar o comportamento”. Então, assim, é uma questão que precisa ser tratada dos dois lados né?* (Enf 16)

### Importância das simulações na formação dos profissionais de saúde

Neste tema, surgiu a importância das simulações para formação dos profissionais de saúde. De acordo com os estudantes de ambos os cursos, a simulação propiciou que os estudantes entrassem em contato com sentimentos, emoções e lembranças que podem interferir no atendimento das mulheres em situação de violência. Nos relatos abaixo, as estudantes compartilharam suas próprias experiências pessoais com a violência, o que demonstra empatia e compreensão pela situação da mulher, além de evidenciar a falta de acolhimento que essas pessoas podem enfrentar tanto pelos profissionais de saúde quanto em outros contextos, como na família.

*Eu só ia compartilhar que foi o meu caso também. No geral, médico, em casa, nada, ninguém nunca foi a fundo. Acho que é uma questão que eu tenho muito próxima e que faz muita diferença, porque eu nunca tive nenhum acolhimento dessa parte, eu enfrentei tudo sozinha, passei sozinha, terminei o relacionamento sozinha e foi isso. Eu resolvi minha questão, mas tem muitas pessoas que não conseguem.* (Enf 8)

*A gente tem que tomar muito cuidado, porque eu já sofri relacionamento abusivo e psicológico, e a violência, ela não se justifica. Gente, só Deus sabe como é difícil sair de um relacionamento abusivo, não é algo fácil, de jeito nenhum, então tem que tomar cuidado.* (Enf 14)

Ficaram evidenciadas a carga emocional que acompanha o atendimento de casos de violência e a necessidade de habilidades socioemocionais, como a capacidade de acolher e compreender as emoções das mulheres. A simulação permitiu que fosse dado lugar ao desconforto em atender tais situações, buscando aprimorar tais habilidades:

*É uma carga emocional muito grande, tem que ter uma habilidade socioemocional para lidar com isso*. (Med 4)

*Eu acho que esses cenários sempre são desconfortáveis, porque a gente consegue perceber o desconforto da paciente, consegue perceber que a todo momento ela está ansiosa. Ela não faz contato visual, então isso gera uma ansiedade na gente.* (Med 23)

Os estudantes destacaram ainda a importância de terem contato com esses casos complexos por meio da simulação antes da prática profissional, uma vez que as simulações oferecem um ambiente seguro e controlado para aprender e praticar, e que lidar com casos reais de violência pode ser emocionalmente difícil e desafiador:

*Acho que aqui é bem mais leve do que na prática realmente. Aqui a gente sabe que é uma simulação, mas, na prática, já apareceu caso de violência e aí você fica meio perdido.* (Med 30)

A fala a seguir destaca a importância das habilidades de comunicação e de lidar com situações difíceis ao atender mulheres em situação de VPI, aspecto que foi trazido à tona com a simulação.

*Acho que é mais de como conversar, como contornar algumas situações, foi o principal, porque a gente normalmente não tem muito contato com essa parte. É muito difícil a gente ser colocado ali de frente com o paciente e ter que lidar com essas situações que são tão estressoras para eles e que a gente fisicamente está vendo que ela está desanimada, tendo sintomas físicos. Ela fala que dorme bem, mas continua muito cansada, então acho que foi o principal.* (Enf 21)

Os estudantes refletiram que a simulação ajuda a *expandir o horizonte* (Enf 11), além de viabilizar uma transição mais suave da teoria para a prática:

*A simulação faz muita diferença em qualquer disciplina que a gente faz, porque, quando a gente chega na prática, a gente já chega com um olhar mais disposto a compreender e a entender o que está acontecendo. Então, eu acho que toda disciplina, se pudesse, deveria ter, porque isso faz com que a gente chegue na prática mais preparados.* (Enf 25)

## DISCUSSÃO

Os estudantes, de ambos os cursos, perceberam a importância de realizar atendimento integral dos casos de violência, abordando questões biopsicossociais. Mobilizaram estratégias de intervenção, como a empatia e estabelecimento de um ambiente de confiança por meio do vínculo, reconhecimento da rede de apoio formal e informal, e cuidados de saúde ao autor de violência. A simulação foi percebida como importante ferramenta na formação dos profissionais de saúde, permitindo que os estudantes tivessem contato com sentimentos e emoções que esses casos trazem antes da prática profissional.

Os estudantes compreenderam a violência como um problema complexo e que trouxe muitas dificuldades em como proceder durante os atendimentos, mas reconheceram a importância de apoiar e vislumbrar o cuidado integral nessas situações. A integralidade como um dos princípios do Sistema Único de Saúde^(16)^ traz a premissa que a assistência à saúde se paute neste conceito. Neste sentido, busca ir além da prática curativa, considerando todos os aspectos individuais e o contexto social, familiar e cultural em todos os níveis de atenção. Há o diálogo ainda com o cuidado centrado no trauma, modelo que vem sendo discutido em especial internacionalmente para indicar caminhos para o cuidado a sobreviventes da violência. Trata-se de modelo centrado na pessoa, sensível e que se dirige a experiências passadas ou presentes de um trauma^(17)^. Permite que as pessoas atendidas retomem seu senso de controle e poder. Busca se apoiar em quatro pilares: reconhecer os sinais e sintomas; aceitar a prevalência e impacto do trauma; enfrentar a revitimização; e responder com políticas e práticas direcionadas à exposição à violência. Entende-se que pessoas enfrentando a VPI preferem não julgamento, compaixão e confidencialidade ao invés de críticas às suas escolhas ou encorajamento para sair da situação^(18)^. É importante destacar que os participantes deste estudo refletiram e manejaram as situações apoiados nesses aspectos.

Revisão de literatura sobre os desafios de se realizar o cuidado diante da violência doméstica na perspectiva dos profissionais de saúde corrobora nossos achados. Existem barreiras relacionadas aos profissionais, como falta de conhecimento e formação, falta de tempo durante o atendimento, falta de confiança para perguntar, julgamento das pessoas, falta de segurança e conforto para perguntar questões sobre violência com outros profissionais próximos^(19)^. Por tudo isso, destacamos a importância de trabalhar o tema da violência contra as mulheres durante a graduação, buscando fornecer subsídios para os futuros profissionais lidarem com esses casos na prática.

Da mesma maneira, outra pesquisa realizada com trabalhadores da APS sobre o atendimento de mulheres em situação de violência doméstica encontrou que os profissionais se sentem inseguros ao abordar o tema. Foi reconhecida a necessidade de serem capacitados para desenvolver competências para realizar esse tipo de abordagem, corroborando mais uma vez nossos achados sobre a importância do tema ser trabalhado com os futuros profissionais^(20)^.

Um aspecto profundamente refletido pelos estudantes de enfermagem, durante as simulações, foi em relação à abordagem e ao cuidado necessário ao autor da violência. Estudo brasileiro buscou verificar a eficácia da Lei “Maria da Penha”, e concluiu que a lei contribuiu para a uma redução de 43% nas hospitalizações relacionadas a agressões contra mulheres, entretanto não impactou atos violentos mais sérios, como injúrias relacionadas a armas brancas ou de fogo. Este fato pode sugerir que os autores continuam a agir com suas tendências agressivas após a promulgação da lei, ou seja, demonstra que o cuidado a esses autores ainda é um desafio. O artigo ainda traz a importância de, para além de estabelecer o empoderamento para que mulher saia da situação de violência, estabelecer na prática uma rede de apoio que garanta a segurança da mesma e de sua família^(21)^. A preocupação diante desses aspectos permeou as discussões dos estudantes de enfermagem, mostrando a mobilização de conhecimentos, bem como maturidade no debate, que foram potenciais para o desenvolvimento de competências cognitivas, atitudinais e procedimentais junto ao tema. Diante do exposto, considera-se fundamental que o cuidado aos agressores deva ser abordado também com os estudantes de medicina, uma vez que esse tema não foi citado por eles durante as simulações.

Ainda sobre o manejo do atendimento, os estudantes de medicina e enfermagem identificaram a importância de dizer para as mulheres sobre os locais ou serviços onde podem buscar ajuda e a importância de elas terem uma rede de apoio. Assim como em nossa pesquisa, estudos recentes, realizados em diversos lugares do mundo, destacam a importância do suporte e apoio social para melhorar a autoconfiança e redução dos efeitos da violência^(22-24)^.

Muitos foram os sentimentos e emoções relatados pelos estudantes ao atenderem casos de violência. Na Espanha, pesquisa com médicos buscou verificar a utilização de protocolos para atendimento à VPI. Os profissionais relataram sentimentos como medo, frustração ou impotência como obstáculos para enfrentar esse problema, sendo fundamental que haja treinamento para que as respostas emocionais dos profissionais não tenham influência negativa no cuidado às mulheres^(25)^. Em nossos achados, tornou-se evidente que tais sentimentos e emoções podem ser discutidos, exercitados e melhor desenvolvidos durante a formação profissional para favorecer melhor atuação prática.

Por conseguinte, permanecendo na discussão sobre sentimentos e emoções, a participação na simulação fez com que algumas estudantes relatassem histórico pessoal de vivência de violência. Geralmente, a fala trazia o sofrimento por lembrar dos casos e as dificuldades pela não compreensão das pessoas, incluindo profissionais de saúde. Trazer à tona sua dor, durante uma simulação, foi um processo importante para essas vítimas/estudantes poderem ajudar mulheres em situação de violência no futuro, durante a prática profissional, por já terem tido contato com seus próprios sentimentos diante desses casos.

Nossos achados deixaram nítido que, para os estudantes, as simulações sobre o atendimento à VPI contra as mulheres são importantes ferramentas de ensino nos cursos de graduação na área da saúde, contribuindo para a abordagem do tema e, consequentemente, deixando os profissionais melhor preparados diante de casos de violência, auxiliando para o fim de todas as formas de violência contra as mulheres. Nosso resultado é corroborado por revisão sistemática de literatura realizada em 2020, na qual a simulação emergiu como estratégia de ensino e aprendizagem que é efetiva no ensino sobre violência^(26)^.

É mister sinalizar a importância do olhar ao cuidado à VPI contra mulheres para alcance dos ODS. Revisão de escopo buscou determinar como os ODS foram integrados, mundialmente, ao ensino superior. Os autores destacaram o quanto os futuros profissionais necessitam de conhecimentos sobre os ODS para orientar suas práticas, com enfoque nos determinantes da saúde. No entanto, encontraram que apenas alguns programas educacionais em saúde integraram explicitamente os ODS, sendo que, em relação ao contexto da igualdade de gênero, nada foi encontrado^(27)^. A presente simulação sobre o atendimento à VPI contra mulher na APS contribui para que esse objetivo elencado pela ONU possa ser alcançado, uma vez que esses profissionais de saúde estarão melhores preparados para lidar com esses casos, auxiliando as mulheres a eliminar todas as formas de violência contra elas. Tal tecnologia pode ser replicada em outros contextos, a um baixo custo e com recursos factíveis.

Finalmente, a importância e relevância da etapa de *debriefing* para o ensino e aprendizagem em enfermagem restou comprovada por meio de revisão de literatura que analisou 140 estudos relacionados a esse tema^(28)^. Esse achado corrobora o que encontramos em nossa pesquisa, pois nossos achados qualitativos foram coletados durante o *debriefing* e demonstraram a importância e riqueza de informações, reflexões e aprendizagens que esta etapa da simulação proporcionou aos estudantes. Ressalta-se que o guia para o *debriefing* holístico se mostrou pertinente e coerente à abordagem deste tema, norteando sistematicamente a discussão para alcance dos objetivos almejados.

### Limitações do estudo

O estudo teve limitações. Realizamos a atividade com estudantes de universidade pública que compareceram voluntariamente à prática de simulação, sendo estudantes dos últimos anos de graduação em medicina e enfermagem que realizaram a atividade de maneira uniprofissional.

Consideramos importante realizar simulações de violência por parceiro íntimo contra as mulheres com estudantes de graduação de outras áreas da saúde, de maneira multiprofissional, de universidades públicas e privadas.

### Contribuições para a área da saúde

Os resultados deste estudo fornecem subsídios para a abordagem da VPI contra as mulheres na APS, para cursos de graduação na área da saúde, por meio de simulações clínicas. Tais tecnologias são factíveis e podem ser transferidas a diferentes contextos. Essa experiência pode contribuir para a melhor formação de profissionais, levando-os a estarem melhor capacitados para o enfrentamento concreto do fenômeno aqui pautado, em consonância com o ODS de alcançar a igualdade de gênero e eliminar todas as formas de violência contra as mulheres.

## CONSIDERAÇÕES FINAIS

Retomando nosso objetivo inicial, a simulação clínica contribuiu para o processo de aprendizagem dos estudantes, que reconheceram a importância do cuidado integral às mulheres em situação de VPI na APS e de exercitarem esse cuidado a partir da simulação. Conhecimentos, habilidades e atitudes foram mobilizados, desenvolvidos e exercitados para a construção deste atendimento, aspecto facilitador para que os estudantes sejam mais capazes no exercício da profissão diante do fenômeno trabalhado.
